# The Effectiveness of Combined Dietary and Physical Activity Interventions for Improving Dietary Behaviors, Physical Activity, and Adiposity Outcomes in Adolescents Globally: A Systematic Review and Meta‐Analysis

**DOI:** 10.1111/obr.13940

**Published:** 2025-05-20

**Authors:** Natalie Pearson, Rebecca Pradeilles, Andrew Kingsnorth, Africa Peral Suarez, Benjamin Boxer, Paula Griffiths, Lauren B. Sherar

**Affiliations:** ^1^ School of Sport, Exercise & Health Sciences Loughborough University Loughborough Leicestershire UK; ^2^ UMR MoISA (Montpellier Interdisciplinary Centre on Sustainable Agri‐Food Systems) Université Montpellier, CIRAD, CIHEAM‐IAMM, INRAE, Institute Agro, IRD Montpellier France; ^3^ Department of Nutrition and Food Sciences Universidad Complutense de Madrid Madrid Spain

**Keywords:** adiposity, adolescents, behavior change, dietary behaviors, physical activity, sedentary behaviors

## Abstract

This systematic review and meta‐analysis examined the effectiveness of combined diet and physical activity interventions on changes in dietary and physical activity behaviors, and adiposity related outcomes in adolescents globally. PubMed, Embase, and Cochrane were searched for controlled interventions targeting dietary behaviors and physical activity in adolescents aged 10–19 years at baseline and reporting on the outcomes of changes in dietary and physical activity behaviors. Behavioral outcomes were synthesized narratively, and meta‐analyses were conducted for changes in adiposity related outcomes (e.g., BMI *z*‐scores, body fat percentage). Thirty‐six studies were included, most (79%) were conducted in high‐income countries and delivered in school settings (*n* = 28, 78%). Ten interventions (28%) showed no effect on any behaviors, and 5 (14%) reported changing all behaviors targeted and assessed. Most (72%) interventions changed at least one of the behaviors assessed, and 39% changed one or more indicator of adiposity. In a subsample (*k* = 16), there was a nonsignificant reduction in BMI (SMD −0.11 [95% CI −0.26 to 0.04]; *I* ^*2*^ = 90%), a significant moderate reduction in BMI *z*‐score (*k* = 14) (SMD −0.62 [−1.09 to −0.16]; *I* ^*2*^ = 99%), and in body fat percentage in favor of the intervention groups (*k* = 11) (SMD −1.32 [−2.22 to −0.42]; *I* ^*2*^ = 99%). The evidence for interventions targeting both dietary and physical activity behaviors and their effect on behavior and adiposity in adolescents is largely inconsistent. The positive findings from few studies suggests that there is potential to improve some lifestyle behaviors and associated adiposity outcomes in adolescents. However, the current evidence is focussed on high income countries with little consideration given to potential inequities in the effects of interventions.

## 1 Background

Over the past four decades, the prevalence of obesity in children and adolescents has increased more than tenfold globally and affects all regions of the world [1]. Obesity puts billions of pounds worth of burden on health services worldwide [2], and this coupled with health consequences for individuals provides strong rational for primary prevention. Adolescence has been identified as a life stage that may play a critical role in the development and persistence of excess weight gain and antecedents for other noncommunicable diseases [3]. Evidence suggests that independence in food and beverage choices increases [4], physical activity decreases [5], and sedentary time increases [6, 7] during adolescence. Therefore, focusing interventions on adolescents has been described as having a potential “triple benefit” through improving the health and wellbeing of adolescents today, into adulthood, and for the next generation [8].

To reduce obesity prevalence, there is a need to better understand the most effective ways of changing the behaviors that are driving obesity in adolescents. Previous systematic reviews have summarized the impact of dietary interventions on changes in dietary behaviors [9] and adiposity related outcomes [10], as well as systematic reviews that summarize the impact of physical activity and sedentary behavior interventions on changes in physical activity [11, 12], sedentary behavior [13], and adiposity related outcomes [14, 15]. These reviews, of mostly school‐based interventions in high‐income countries, suggest mixed evidence on the effectiveness of interventions to improve individual behaviors [11, 15, 16] and marginal impact on adiposity outcomes [10, 15, 17]. Given that most adolescents engage in multiple unhealthy behaviors that place them at increased risk of poor health [18, 19], targeting multiple health behaviors, such as dietary behaviors and physical activity/sedentary behavior together, may be more effective at changing behavior and adiposity‐related outcomes [20, 21]. However, to date, there has been a lack of systematic reviews of combined dietary and physical activity/sedentary behavioral interventions that report on changes in behavior as opposed to their effects on obesity related outcomes only [20, 21]. In a 2005 Cochrane review, findings from 14 youth obesity prevention studies that targeted physical activity and dietary change were summarized [22], with only one study reporting effectiveness at changing both dietary and physical activity behaviors, and this was only among girls. Updates of such reviews have reported on adiposity related outcomes and have not included evidence on the effect on changes in dietary and physical activity behaviors [20, 23]. This gap in the evidence limits our understanding of what works best to change these complex behaviors that are driving obesity rates. Furthermore, most reviews of behavioral interventions focus on effectiveness with few providing an evaluation of some of the key components of interventions [24] or reporting on information surrounding the equity of an impact of an intervention (where an intervention may not be equally benefiting subgroups of individuals within the population) [12, 25]. This is essential information as interventions can in fact contribute to widening inequalities in health and health behaviors [12], due to, for example, the implementation, access, uptake, and compliance of interventions [26]. Detailing the active ingredients and intervention features of combined diet and physical activity interventions will help build cumulative evidence towards delivering effective replicable interventions to positively change behavioral and obesity related outcomes. This systematic review, therefore, has a primary aim of synthesizing the evidence on the effectiveness of interventions targeting both diet and physical activity on changes in diet and physical activity behaviors among adolescents globally. A secondary aim is to examine the effect of such interventions on adiposity‐related outcomes if and where reported and to explore any equity effects, strategies, and key components of interventions that contribute to effectiveness.

## 2 Methods

This systematic review was registered with the International Prospective Register for Systematic Reviews ((PROSPERO) CRD42022315551) and is reported according to the Preferred Reporting Items for Systematic Reviews and Meta‐Analyses (PRISMA) statement [27].

### 2.1 Eligibility Criteria

We considered studies to be eligible for inclusion if they had conducted an intervention study with a usual practice control/comparator group (e.g., randomized controlled trials; nonrandomized controlled trials; pre/poststudies with a control), comprised adolescent participants aged between 10 and 19 years at baseline of the study, and evaluated combined dietary and physical activity interventions that reported quantitative data related to change (from pre to postintervention/follow‐up) in any domain of physical activity and change in any domain of dietary behavior. Table 1 lists the inclusion and exclusion criteria.

**TABLE 1:** Study inclusion and exclusion criteria.

	Inclusion criteria	Exclusion criteria
Study aim	Behavior change	Weight loss/change in weight related outcomes only
Population	Adolescents aged 10–19 years of age	Studies that exclusively enrolled participants with a disease or clinical populations. Samples of children < 10 years or adults > 19 years
Setting	Any setting	
Interventions	Behavioral interventions with a focus on targeting and changing physical and dietary behaviors. Interventions could be delivered in any means (e.g., face‐to‐face, online, or using technology). There was no restriction on who delivered the interventions (e.g., teachers, researchers)	Interventions designed specifically for the treatment of childhood obesity and RCTs designed to treat eating disorders such as anorexia and bulimia nervosa. Interventions that did not target and both physical activity and diet as intervention components.
Comparisons	No intervention (e.g., wait list control, usual care); attention control (e.g., similar format and intensity to intervention but different content area (e.g., focus on sun care or different health behavior)	Active comparators without a control
Outcomes	Any quantitative measure of physical activity and any quantitative measure of dietary behavior. We also included anthropometry (e.g., weight, BMI) and sedentary behavior related outcomes only if they had both physical activity and dietary behavior outcomes	Only reported anthropometry related outcomes
Timing of assessment	Include data at baseline and postintervention/follow‐up of any length intervention	

### 2.2 Search Strategy

Searches of electronic databases (PubMed, Embase, and Cochrane) were conducted between February 2022 and July 2023. The search strategy was developed using the population, intervention, comparison/control, outcome (PICO) model: population (adolescents aged 10–19 years), intervention (combined dietary and physical activity interventions with a C: comparison/control group), and behavioral outcomes (e.g., any quantitative outcomes of physical activity and dietary behaviors). Supplementary searches were conducted that included manual searches of personal files, and screening reference lists of primary studies and identified review articles (e.g., [20]) for titles that included the key terms. Each of the three databases were searched using database‐specific indexing terms. The search syntax was first developed for PubMed and then adapted to the database‐specific search requirements. Search strategies are provided in Supporting File 1. No date limitations were applied to the searches. While data on sedentary behavior and adiposity related outcomes were extracted where reported (see below) as secondary outcomes, sedentary behavior and adiposity related keywords were not part of the search strategy because they were not the primary focus of this review.

### 2.3 Identification of Relevant Studies

Covidence review management software (www.covidence.org) was used to manage this review. Results identified from the search strategies were uploaded to Covidence, where all duplicates were removed. Two independent reviewers from BB, NP, and APS initially screened the titles and abstracts for eligibility and identified studies for full text review. Two reviewers from BB, NP, and APS independently accessed and screened the full texts of studies against the inclusion criteria to determine eligibility. A third reviewer (either NP or RP) assessed a random sample of 10% of the excluded studies at both title/abstract and full text stages. Disagreements were discussed and resolved with a fourth reviewer (either NP or RP). All decisions for inclusion and exclusion were recorded in Covidence, and reviewers were blinded to each other’s decisions.

### 2.4 Data Extraction

Data extraction forms were developed specifically for this review in Microsoft Excel. Two reviewers (B.B. and N.P.) completed the data extraction for all included studies, and a sample of papers (10%) were checked by third and fourth reviewers (R.P. or A.P.S.) for completeness.

Data were extracted on the characteristics of included studies: (i) general information (study ID, title, authors, date, study location (country, level of income of country according to World Bank Classification, urban vs. rural), study aim); (ii) study eligibility (participant selection and randomization process (for randomized studies), sample size, participant characteristics), type and duration of intervention, setting of intervention (e.g., school, community), intervention components and intervention strategies (i.e., active ingredients, intervention features), and theories utilized (e.g., social cognitive theory (SCT)); (iii) methods and measures of the behaviors of interest; and (iv) results for outcomes of interest (estimates, list of confounders, narrative summary of results, study limitations). The primary outcomes were changes in physical activity and dietary behaviors, and where measured/reported, we also extracted data on changes in sedentary behaviors and change in any adiposity related outcomes (e.g., BMI *z*‐scores, body fat percentage (%)). Following standard procedures, data on outcomes of interest at baseline and postinterventions (first follow‐up) were extracted [20].

As one of the aims of this review was to identify interventions that had reported on indicators important from an equity perspective, information relevant to equity was extracted using the PROGRESS‐Plus framework [28, 29]. Given that this review focused on adolescents, data on targeting of interventions and differential effects were considered across the PROGRESS‐Plus framework applicable to adolescents: gender, socioeconomic status, ethnicity, place of residence, and religion.

### 2.5 Risk of Bias (RoB) and Evidence Assessment

A RoB assessment was completed for each study. For RCTs, the Cochrane RoB‐2 was used [30]. Two reviewers (B.B. and N.P.) independently assessed each study against each of the five domains and rated them as low, some concerns, high RoB, or no information [30]. For non‐RCTs, ROBINS‐I was used [31]. Two reviewers (B.B. and N.P.) independently assessed each study against each of the seven domains and rated them as being at low, moderate, serious, or critical RoB or no information [31]. Supporting information and justifications for judgments in each domain were recorded for all studies. A third reviewer (R.P. or A.P.S.) compared ratings, discussed discrepancies, and agreed on the overall RoB, which was assessed using the Cochrane guidance.

### 2.6 Outcomes and Evidence Synthesis

The primary outcomes were changes in dietary behaviors and physical activity, and where reported, changes in sedentary behaviors were also extracted and synthesized. Dietary, physical activity, and sedentary behavior outcomes were extracted as per the reporting in individual studies. Heterogeneity arose across studies based on methods, measures/units and outcomes of physical activity and dietary outcomes, which precluded meta‐analyses of these behaviors. While data on mean differences in all behavioral outcomes was extracted where available, the data were synthesized narratively. The effect of the interventions on each physical activity, dietary, and sedentary behavior outcome was coded as follows: ↑*: “positive and statistically significant effect” (i.e., there was an increase in physical activity or fruit consumption and in favor of the intervention group), ↓*: “negative and statistically significant effect” (i.e., a decrease in screen time or sugar‐sweetened beverage (SSB) consumption in favor of the intervention group), or 0: “no statistically significant effect” (i.e., no statistically significant difference in the outcome between the intervention and control group). All identified dietary, physical activity, and sedentary behavior outcomes are displayed in Supporting File 2 as described in the individual studies, but for brevity, all dietary behaviors were classified as favorable (e.g., consumption of fruit, vegetables, not skipping breakfast) and unfavorable (e.g., consumption of SSBs, sweet/salty snacks, fast foods etc, skipping breakfast), and all domains of physical activity (e.g., walking, steps, moderate vigorous physical activity (MVPA)) and sedentary behavior (e.g., sitting time, screen‐time) were classified as “physical activity” and “sedentary behavior” respectively and synthesized narratively using the codes ↑*, ↓*, and 0 as described above. Each individual outcome behavior was counted per study; for example, if a study reported on four favorable dietary outcomes, then the summary table will have four codes (e.g., ↑*, ↑*, ↓*, and 0) in the column favorable dietary behaviors.

Data on changes in adiposity related outcomes were included as an important secondary outcome when/if reported in addition to changes in the primary outcomes of interest. Meta‐analyses were conducted using the meta package Version 6.5–0, Schwarzer (2023) in R (Version 4.3.1) for the outcomes BMI, BMI *z*‐scores and body fat percentage, as these were the outcomes most frequently reported across studies. We used random effects models as we expected heterogeneity in the intervention effects because of the differences in study populations and the diversity of intervention components and comparisons. We calculated the standardized mean difference (SMD) change in adiposity outcomes using Hedges’ g effect size with 95% confidence intervals [32]. Pooled mean difference and variance in the heterogeneity between studies (*I* ^2^) was calculated and presented using forest plots. Where studies had more than one intervention group, we divided the number of participants in the control group by the number of intervention groups and analyzed each individually.

Interventions included different components from targeting education (e.g., knowledge and active learning), targeting the social environment (e.g., including parents), and targeting the physical environment (e.g., environmental changes) and used a range of different strategies and behavior change techniques (BCTs) to support changes in physical activity and dietary behaviors. Intervention components and strategies reported in studies are described in Supporting File 3, alongside a summary of the effect of each intervention on changes in dietary behaviors, physical activity, sedentary behavior, and indicators of adiposity.

Equity data were summarized using graphical and narrative methods to describe whether studies had gathered equity data at baseline and whether they had subsequently conducted any equity analyses.

## 3 Results

**FIGURE 1:**
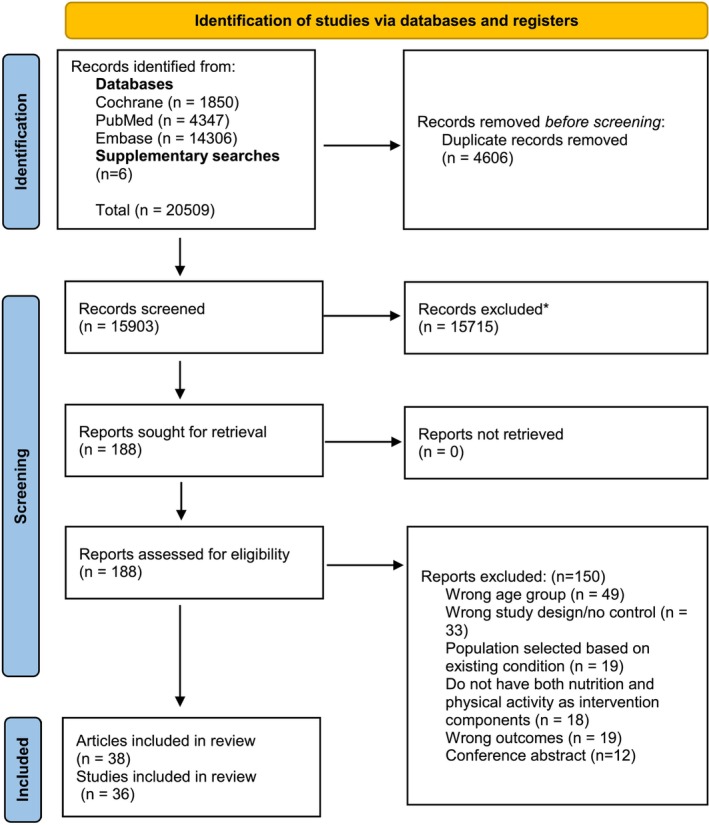
Flow chart of search strategy.

The literature searches yielded 20,509 titles of potentially relevant articles, of which 38 articles of 36 studies were considered eligible for this review (see Figure 1).

### 3.1 Characteristics of Included Interventions

The characteristics of included interventions are described in Table 2. Most interventions were RCTs (*n* = 29, 81%), and most (69%) were conducted in high‐income countries (HICs) within Europe (*n* = 11) and the United States (*n* = 9), followed by Australia (*n* = 3), Canada (n = 1), and Trinidad and Tobago (n = 1). Eleven interventions (31%) were conducted in middle‐income countries (MICs): Two interventions conducted in each of Brazil and Turkey, and one in each of Argentina, Vietnam, Fiji, South Africa, Lebanon, Tonga, and Thailand. Most studies (*n* = 28, 78%) had intervention components that were delivered solely in the school setting, one in school plus community and one in school plus home. The remaining studies were delivered in university residence (*n* = 1), university plus home (*n* = 1), community‐based settings (*n* = 2), and in a primary health care setting (*n* = 1). Interventions ranged from 2 × 50 min sessions to 3 years in duration, with more than half of interventions (*n* = 20, 56%) being between 6 and 24 months.

**TABLE 2:** Characteristics of intervention studies included in systematic review and meta‐analysis (*n* = 36) according to intervention components.

Study (country)	Baseline age (mean or range) in years	Gender and baseline n	Setting	Theory	Duration	Intervention behavior targeted		Outcome methods and measures		
						Dietary target	Physical activity (PA)/sedentary behavior target	Dietary method and measure	PA method and measure	Anthropometry indicators
**Educational components only**										
Ardic et al. 2017 [33] (Turkey)	I: 12.8 years C: 12.8 years	GB (I:45, C:42)	School	NS	15 weeks	Water and FV consumption	Walking	SRQ: mL, meals/day	Pedometer: steps/day	Self‐report: BMI kg/m^2^
Contento et al. 2010 [34] (USA)	12 years	GB (I: 562, C:574)	School	SDT SCT	8–10 weeks	Decreasing SSB, packaged snacks, fast food. Increasing water, FV.	Decreasing leisure ST: Increasing PA	SRQ: quantity and frequency of consumption	SRQ: days/week	Not measured
Epton et al. 2014 [35] (UK)	I: 19.0 years C: 18.9 years	GB (I:736, C:709)	University	TPB	Online intervention (duration unclear) FU at 1 and 6 months	FV	PA Sedentary time	SRQ: Portions/day	IPAQ: METs/week	Self‐report: BMI kg/m^2^
Fairclough et al. 2013 [36] (UK)	10–11 years	GB (I: 166, C: 152)	School	SCT	20 weeks	Healthy eating	Increasing PA: reducing sedentary/technology time	24‐h recall: previous day consumption	Actigraph: m/day	Measured: BMI kg/m^2^: WC
Francis et al. 2010 [37] & Nichols et al. 2014 [38] (Trinidad and Tobago)	C: 10.6 I: 10.2	GB (I:280, C:299)	School	Blooms mastery learning model	One month (FU at 3 and 18 months)	Dietary behavior	Improve PA habits/reduce TV viewing	FFQ: Frequency over last week and last 24 h	SRQ: hours/day	Measured: BMI kg/m^2^
Frenn et al. [39] 2005 (USA)	12–14	GB (I:43, C:60)	School	HPM TTM	8 sessions	Reduce dietary fat consumption	Increase PA	SRQ: % energy intake	SRQ: min/week	Not measured
Rutsztein et al.2023 [40] (Argentina)	I: 15.13 years C: 15.59 years	G (I: 230, C: 279)	School	CDT	3 weeks	Promote healthy eating practices and reduce skipping breakfast	Well‐being (movement/PA)	SRQ: days/week	SRQ: days/week	Self‐report: BMI kg/m^2^
Weigensberg et al. 2021 [41] (USA)	16.4 years	GB (I:181, C:51)	School	NS	12 weeks	Dietary behavior	Reducing sedentary behavior, increase light activity, moderate activity, vigorous activity	Multiple pass DRI: kcal/day, g/day	Accelerometer: m/day	Not measured
**Educational and social environmental components**										
Akdemir et al. 2017 [42] (Turkey)	I: 10.6 years C: 10.2 years	GB (I: 674, C: 675)	School	NS	1 academic year	Improving diet quality	Increasing walking: reducing computer games/TV viewing	SRQ: KIDMED index, frequency of consumption	SRQ	Measured: BMI kg/m^2^
Angelopoulos et al. 2009 [43] (Greece)	I: 10.25 C: 10.29	GB (I:321, C:325)	School	TPB	12 m	Increase consumption of FV	PA during leisure time	24 h recall: exchanges/day	SRQ: m/day	Measured: BMI kg/m^2^
Champion et al. 2023 [44] (Australia)	12.7 years	GB (I: 3610, C: 3030)	School	NS	Duration unclear. FU at post‐intervention, 12 months, and 24 months	SSB	MVPA, screen time	SRQ: frequency/week	SRQ: d/week, h/day	Not measured
Ezendam et al. 2012 [45] (Netherlands)	I: 12.7 years C: 12.6 years	GB (I: 485, C:398)	School	TPB PAPM	8 lessons over 10 weeks (FU at 4 and 24 months)	Healthy eating	Increasing PA. Decreasing sedentary behavior	FFQ: Times/week, times/day	SRQ: times/week, duration/week, meeting MVPA guidelines.	Measured: BMI kg/m^2^
Jemmott III et al. 2019 [46] (South Africa)	12.4 years	GB (I:495, C: 562)	School	SCT TPB	12 x 1 h intervention sessions	Adherence to 5‐a‐day diet, reduction of fried foods	Meeting PA guidelines	FFQ: serv/day, meeting intake guidelines	SRQ: days/week and %	Not measured
Pablos et al. 2018 [47] (Spain)	I: 10.7 years C: 10.6 years	GB (I:82, C:76)	School	NS	8 m	Healthy lifestyle	Healthy lifestyle	SRQ: %, index	SRQ: m/week, h/day	Measured: BMI kg/m^2^
Patrick et al. 2006 [48] (USA)	I: 12.7 years C: 12.7 years	G (I:222, C:216) B (I:202, C:179)	Primary health care clinics	BDM SCT TTM	12 m	Intake of FV, fiber and total dietary fat	Moderate or vigorous PA. Sedentary behavior	Three 24‐h dietary recalls: g/day, servings/day, % calories	7‐day Physical Activity Recall: m/week, d/week	Measured: BMI kg/m^2^
Prieto Zambrano et al. 2021 [49] (Spain)	12.78 years	GB (I:46, C:36)	School	NS	2 × 50 mins	Quality of the Mediterranean diet	Degree of PA	KIDMED Test	PAQ‐A	Self‐report: BMI kg/m^2^
Sevil et al. 2019 [50] (Spain)	13.1 years	GB (I:225, C:115)	School	SEM SDT TPB	1 school year	Water and healthy food consumption	PA promotion	SRQ: % meeting guidelines	Accelerometer: m/day, %meeting guideline	Self‐report: BMI kg/m^2^
Sgambato et al. 2019 [51] (Brazil)	I: 11.5 years C: 11.5 years	GB (I:1290, C:1157)	School and home	NS	6 × 50 min sessions	Reducing intake of cookies and SSBs, processed foods. Encouraging FV & water consumption.	Increasing PA and reducing sedentary behavior	FFQ: glasses/ladles/units/tablespoons/packages/slices	SRQ: m/week	Measured: BMI kg/m^2^
Spiegel et al. 2006 [52] (USA)	4^th^/5^th^ grade	GB (I:529, C:478)	School	TRA	6 m	FV consumption	PA participation at school and outside school	SRQ: consumption	SRQ: m/day, m/week	Measured, BMI kg/m^2^
Thi Nguyen et al. [53] (Vietnam)	I: 11.3 years C: 11.8 years	G (I: 69, C:56) B (I: 69, C: 69)	School	NS	7 m	Promote healthy nutritional intake (total energy, protein, fat, carbohydrate, FV, sweet foods)	Increase MVPA time and reduce sedentary time	FFQ: kcal/g/day	SRQ: m/day	Measured: BMI kg/m^2^
**Educational and physical environnemental components**										
Barbosa‐Filho et al. 2019 [54] (Brazil)	11–18 years	GB (I:548, C:537)	School	SCT SEM	4 m	Healthy and unhealthy eating habits	PA, TV viewing, computer use	SRQ: Frequency	SRQ: time/week	Not measured
Chawla et al. 2017 [55] (Thailand)	I: 9.73 years C: 10 years	GB (I: 490, C:490)	School	HBM	6 m	Healthy eating	Being physically active	SRQ: Consumption	SRQ: h/week	Measured: BMI kg/m^2^
Millar et al. 2011 [56] (Australia)	I: 14.5y C: 14.7 years	GB (I:1852, C:1188)	School	NS	3 years	Promoting water consumption and healthy breakfasts, reducing SSB consumption	Promoting active transport to/from school: increasing participation in organized sports and other active recreation	SRQ: Serv/day, %	SRQ: times/week, %	Measured: BMI kg/m^2^
Tarro et al. 2019 [57] (Spain)	9‐13 years	GB (I:375, C:327)	School	Social marketing principles	10 m	Fruit consumption ≥ 1/day	PA ≥ 4 h/week	SRQ: %	SRQ: %	Not measured
**Educational, social, and physical environmental components**										
Aceves‐Martins et al. 2022 [58] (Spain)	I: 14.5 years C: 14.4 years	G (I:83, C:117) B (I:86, C:106)	School	Yes (NS)	12 months	Healthy eating (increase FV & breakfast)	Health lifestyles (increasing PA: reducing screen‐time)	SRQ (portions/frequency of consumption)	SRQ: h/week	Self‐report: BMI kg/m^2^
Baltaci et al. 2022 [59] (USA)	I: 12.2 years C:12.2 years	GB (I: 77, C: 70)	Community	SCT	8 weeks	Healthy eating (increase FV, reduce SSB, fast food, sweet/salty snacks)	Active lifestyles (increase PA, reduce screen‐time)	24 h recall: servings/day	SRQ: h/day	Measured: BMI kg/m^2^
Brown et al. 2013 [60] (USA)	10–14 years	GB (I: 38, C:38)	School and community	TTM SCT	3 m	Lowering fat intake	Increasing PA	24‐h recall: %	Actical: m/day	Measured: BMI kg/m^2^
Brown et al. 2014 [61] (Canada)	17.9	GB (I:174, C: 174)	University halls of residence	SCT	1 academic year	Increase FV consumption	MVPA	SRQ: Frequency consumption	SRQ: MVPA	Not measured
Fotu et al. 2011 [62] (Tonga)	I:14.4 years C: 15.2 years	GB (I:1083, C:1396)	Community	NS	Unclear duration: FU 2.5 years	Eating breakfast, increasing water and fresh FV intake. Reducing SSB.	Participation in organized sports and PA during and after school. Reducing sedentary activities	SRQ: time/week, times/day	SRQ: times/week	Measured: BMI kg/m^2^
French et al. 2011 [63] (USA)	Not stated but > 12	GB (I: 43, C:44)	University and home	NS	12 m	Limiting high calorie snacks, replacing prepackaged meals, limiting SSB and fast‐food, increasing FV intake	Reducing TV viewing, increasing physical activity	FFQ: serv/day	IPAQ and SRQ: m/day	Measured: BMI kg/m^2^
Habib‐Mourad et al. 2020 [64] (Lebanon)	I: 9.8 years C: 10.1 years	GB (I:698, C: 541)	School	SCT	24 m	Healthy dietary behaviors	Active lifestyle	SRQ: times/day	SRQ: times/week	Measured: BMI kg/m^2^
Haerens et al. 2006 [65] (Belgium)	13.1 years	GB (Ia:1226, Ib:1006, C:759)	School	TTM TPB	24 m	Healthy eating	Increasing levels of MVPA to ≥ 60 min a day	FFQ: g/day, %, pieces/week, glasses/day	SRQ: m/day	Measured: % overweight
Kremer et al. 2011 [66] (Fiji)	I: 15.4 C: 15.2	GB (I: 2670, C: 4567)	School	NS	24 m	Healthy eating	Regular PA	SRQ: serv/day, consumed or not %	SRQ: % reporting behavior	Self‐report: BMI kg/m^2^
Lubans et al. 2012 [67] & Dewar et al. 2013 [76] (Australia)	13.2 years	G (I:178, C:179)	School	SCT	12 m	Improve dietary intake	Promote lifestyle and lifetime PA	SRQ: kJ/day	Accelerometer: counts/min	Measured: BMI kg/m^2^
Vieira et al. 2021 [68] (Portugal)	I: 11.2 years C: 11.2 years	GB (I:240, C:264)	School	TTM	8 m	Healthy eating	Active living	FFQ: serving size/day	SRQ: m/day	Measured: WC (cm), BMI kg/m^2^
Williamson et al. 2012 [69] (USA)	10.5 years	GB (I1: 612, I2: 638, C: 447)	School	NS	28 m	Healthy eating (increase FV, reduce dietary fat, increase fiber intake.	Reduce sedentary behavior: increase PA	Digital photograph: Kcal	SAPAQ: h/day	Measured: body fat, BMI kg/m^2^

Abbreviations: B, boys; BDM, behavioral determinants model; BMI, body mass index; C, control; CDT, cognitive dissonance theory; DRI, dietary recall interview; FFQ, food frequency questionnaire; FV, fruit and vegetables, G, girls; HBM, health belief model; HPM, health promotion model; I, intervention; MVPA, moderate, to, vigorous physical activity; PA, physical activity; PAPM, Precaution Adoption Process Model; SCT, social cognitive theory; SDT, self, determination theory; SEM, socioecological model; SRQ, self‐report questionnaire; ST, screen time; TPB, theory of planned behavior; TRA, theory of reasoned action; TTM, transtheoretical model; WC, waist circumference.

Twenty‐three interventions (64%) outlined clear theoretical underpinnings, and 39% of those outlined the use of more than one theory. All studies apart from two, which recruited only girls, included both boys and girls in their interventions. Five interventions (14%) included participants aged 15 years or older at baseline, 30 (83%) included participants aged 15 years or younger, and one study reported participants with an age range of 11–18 years at baseline. Group sample sizes ranged from 36 to 4567.

Most interventions (*n* = 28, 78%) targeted and measured more than one dietary behavior (e.g., decreasing SSBs and increasing fruit and vegetable consumption), 10 interventions (28%) targeted and measured more than one physical activity behavior (e.g., increasing walking and increasing MVPA) as intervention outcomes. In addition, 26 studies (72%) targeted and measured at least one outcome of sedentary behavior, with six studies (23%) targeting and measuring more than one sedentary behavior. Twenty‐seven different dietary behavioral outcomes, 10 physical activity outcomes, and five sedentary behavior outcomes were reported across included studies (see Supporting File 2). Outcome behaviors (physical activity, dietary, and sedentary behavior) were mostly measured with self‐report tools, with five studies (14%) using accelerometers to measure physical activity, and one using a pedometer to measure step count. Twenty‐eight studies measured at least one indicator of adiposity at baseline, with all 28 studies measuring height and weight via either self‐report (*n* = 8, 28%) or by trained staff (*n* = 20, 72%) (Table 2). Overall, of the 29 RCT studies, 37.5% (*n* = 11) presented a high‐RoB summary score, and 62.5% (*n* = 18) presented some concerns. Of the seven non‐RCTs, 29% (*n* = 2) presented serious RoB summary score, and five studies (71%) presented a moderate risk (see Figures 2a–d in Supporting File 4).

### 3.2 Intervention Effects on Primary Outcomes: Physical Activity and Dietary Behaviors

Eight studies (22%) included educational components only, 12 (33%) included educational plus social environmental components, 4 (12%) included educational plus physical environmental components, and 12 (33%) included educational, social, and physical environmental components (Table 3).

**TABLE 3:** Summary level description of intervention effects on dietary behaviors, physical activity, sedentary behavior, and indicators of adiposity.

Study by intervention type	Dietary behavior outcomes		Physical activity/sedentary outcomes		Indicators of adiposity
	Unfavorable dietary behaviors (e.g., sugar sweetened beverage consumption)	Favorable dietary behaviors (e.g., fruit and vegetable consumption)	Physical Activity (e.g., active travel)	Sedentary behavior (e.g., television viewing)	
**Educational components only**					
Ardic et al. 2017 [33]		↑*, ↑*	↑*		↓* weight 0 BMI
Contento et al. 2010 [34]	↓*, ↓*, ↓*, ↓*	0, 0, 0, 0, 0, 0	↑*, ↑*	↓*	NM
Epton et al. 2014 [35]		0	0	0	0 BMI
Fairclough et al. 2013 [36]		0, 0, 0	0, 0, 0	0	↓* WC^33^ 0 BMIz 0 BMI
Francis et al. 2010 [37] & Nichols et al. 2014 [38]	↓*, ↓*, ↓*	0, 0	0, 0	0	NR
Frenn et al. 2005 [39]	↓*		↑*		NM
Rutsztein et al.2023 [40]	↓*		0		NR
Weigensberg et al. 2021 [41]	0, 0, 0, 0, 0, 0	0, 0	0, 0, 0, 0	0	NM
**Educational and social environmental components**					
Akdemir et al. 2017 [42]	↓*, ↓*, ↓*, ↓*	↑*, ↑*, ↑*, ↑*	↑*	↓*	↑* BMI ↓* obesity prevalence
Angelopoulos et al. 2009 [43]	↓*, ↓*, ↓, ↓*	↑*, 0	↑*		↓* BMI 0 BMIz
Champion et al. 2023 [44]	0		0	0	NM
Ezendam et al. 2012 [45]	↓*, ↓*	↑*, 0, 0	0, ↓*	0	0 BMI 0 obesity prevalence 0 WC
Jemmott III et al. 2019 [46]	↓*	↓*, ↓*	↓*, ↓*, ↑*		NM
Pablos et al. 2018 [47]		0, 0	0	0	↓* obesity prevalence 0 BMI
Patrick et al. 2006 [48]	B: 0 G: 0	B: 0 G: ↑*	B: 0, ↑* G: 0, 0	B: ↓* G: ↓*	G: 0 BMIz B: 0 BMIz
Prieto Zambrano et al. 2021 [49]	0, 0, 0, 0	0, 0	0		NR
Sevil et al. 2019 [50]	B: ↓* G: ↓*	G: ↑*, ↑* B: ↑*, ↑*	G: ↑*, ↑* B: ↑*, ↑*	B: ↓*, ↓* G: ↓*, 0	NR
Sgambato et al. 2019 [51]		G: ↑* B: 0	G: ↑* B: ↑*		G: 0 BMI, 0 %BF B: 0 BMI, ↓* % BF
Spiegel et al. 2006 [52]		↑*	↑*		↓* BMI percentile ↓* Obesity prevalence
Thi Nguyen et al. 2022 [53]	↓*, 0, 0, 0, 0	0, 0	0	0	NR
**Educational and physical environmental comonents**					
Barbosa‐Filho et al. 2019 [54]	0, 0, 0	0, 0	↑*	↑*	NM
Chawla et al. 2017 [55]	0, 0	0, 0	0	0	0 Obesity prevalence
Millar et al. 2011 [56]	0, 0, 0	0, 0, 0	↑*	↑*, ↓*	↓* obesity prevalence ↓* BMIz 0 BMI 0 %BF
Tarro et al. 2019 [57]	B: 0, 0 G: ↑*, 0	B: 0, 0 G: 0, 0	B: 0 G: 0	B: ↑*, 0 G: 0, 0	NM
**Educational, social, and physical environmental comonents**					
Aceves‐Martins et al. 2022 [58]		B: 0, 0, 0 G: 0, 0, 0	B: ↑* G: 0	B: 0 G: 0	B: 0 (obesity prevalence) G: 0 (obesity prevalence)
Baltaci et al. 2022 [59]	0, 0, 0	0, 0	0	0	0 BMI percentile
Brown et al. 2013 [60]	0, 0, 0		0	0, 0	0 BMI 0 BMIz
Brown et al. 2014 [61]		0	↑*		NM
Fotu et al. 2011 [62]	↑*, ↓*, ↑*, 0	0, 0, ↓*, ↑*	↓*, ↓*	0, 0, 0	↓* %BF (G&B) 0 BMI 0 BMIz 0 obesity prevalence (G&B)
French et al. 2011 [63]	0, 0, 0	↑*	0	0	0 BMIz
Habib‐Mourad et al. 2020 [64]	↓*, ↓*, ↓*	↑*, ↑*	0		0 obesity prevalence 0 BMIz
Haerens et al. 2006 [65]	B: 0, 0 G: 0, ↓*	B: 0, 0 G: 0, 0	B: ↑*, ↓* G: 0, ↓*	B: 0 G: 0	NR
Kremer et al. 2011 [66]	0, 0, 0	↑*, 0, 0	0	↑*, 0	↓* % BF, 0 BMI 0 BMIz 0 obesity prevalence
Lubans et al. 2012 [67] & Dewar et al. 2013 [76]	0		0	↓*	0 %BF 0 BMI 0 BMIz
Vieira et al. 2021 [68]	↓*, 0, 0, 0	0	↑*	0	B: 0 BMI, ↓* WC G: 0 BMI, ↓* WC 0 BMIz
Williamson et al. 2012 [69]	0, 0, 0, 0, 0		0	0	B: 0% BF, 0 BMIz G: 0% BF, 0 BMIz

*Note:* ↑*: positive (increase) relationship and significant association. ↓*: negative (reduction) relationship and significant association: 0: no significant differences between intervention and control groups. Not reported refers to studies where height and weight were measured for descriptive purposes only. Not measured refers to studies where height and weight were not measured as part of the study. *Full details of individual behavioral outcomes are detailed in the supporting tables.

Abbreviations: %BF: body fat percentage; B: boys; BMI: body mass index; BMIz: body mass index z‐scores; G: girls; WC: waist circumference.

### 3.3 Educational Only Interventions

Of the eight interventions that included educational components only, 2 (25%) were effective at positively changing all behaviors targeted and assessed [33, 39]. Of these two, one was effective at reducing weight but not BMI [33], and one did not measure indicators of adiposity [39]. Three educational interventions had no effect on any behaviors targeted and assessed [35, 36, 41]; of these, one had no effect on BMI [35], one was effective at reducing waist circumference but not BMI [36], and one did not measure indicators of adiposity [41]. Three interventions showed mixed results [34, 37, 40]. None of these three studies reported on indicators of adiposity (Table 3).

### 3.4 Educational and Social Environmental Interventions

Of the 12 interventions that included educational plus social environmental components, three (25%) were effective at changing all behaviors targeted and assessed [42, 46, 52], of which one did not report data on indicators of adiposity [46], one reduced both BMI and obesity prevalence [52], and one increased BMI and reduced obesity prevalence [42]. The interventions effective at changing behavior included home components such as homework or newsletters to parents. Five interventions (42%) were effective at changing most behaviors targeted and assessed, with difference seen between subgroups (i.e., effective in boys but not girls) and within behaviors (i.e., changing unfavorable but not favorable dietary behaviors) [43, 45, 48, 50, 51]. Of these, two were not effective at changing any indicators of adiposity assessed [45, 48], one did not report on indicators of adiposity [50], and two were effective at changing some indicators but not all [43, 51] (Table 3).

### 3.5 Educational and Physical Environmental Interventions

Of the four interventions that included educational and physical environmental components, one showed no effect on behaviors or obesity prevalence [55], and one found no effect on favorable dietary behaviors and physical activity but reported gender differences in unfavorable dietary behaviors and sedentary behaviors [57]. Two reported changes in physical activity and sedentary behavior but no effect on dietary behaviors, one of which did not measure anthropometric indicators [54], and one reported reductions in obesity prevalence, BMI *z*‐scores, but no change in BMI or body fat percentage [56] (Table 3).

### 3.6 Educational, Social, and Physical Environmental Interventions

Of the 12 interventions that included educational, social, and physical environmental components, 3 (25%) showed no effect on any behavior targeted and assessed and no effect on indicators of adiposity [59, 60, 69]. One intervention had a positive effect on all dietary behaviors assessed but not effect on physical activity or BMI *z*‐scores and obesity prevalence [64]. The remaining eight interventions showed mixed effects with little consistency across studies. Of these 8 studies, 4 (50%) had no effect on any indicators of adiposity assessed [58, 63, 67, 68], 2 (25%) did not report on indicators of adiposity [61, 65], and 2 (25%) showed reductions in body fat percentage but not in BMI or obesity prevalence [62, 66] (Table 3).

### 3.7 Meta‐Analysis of Secondary Outcomes: Markers of Adiposity

**FIGURE 2:**
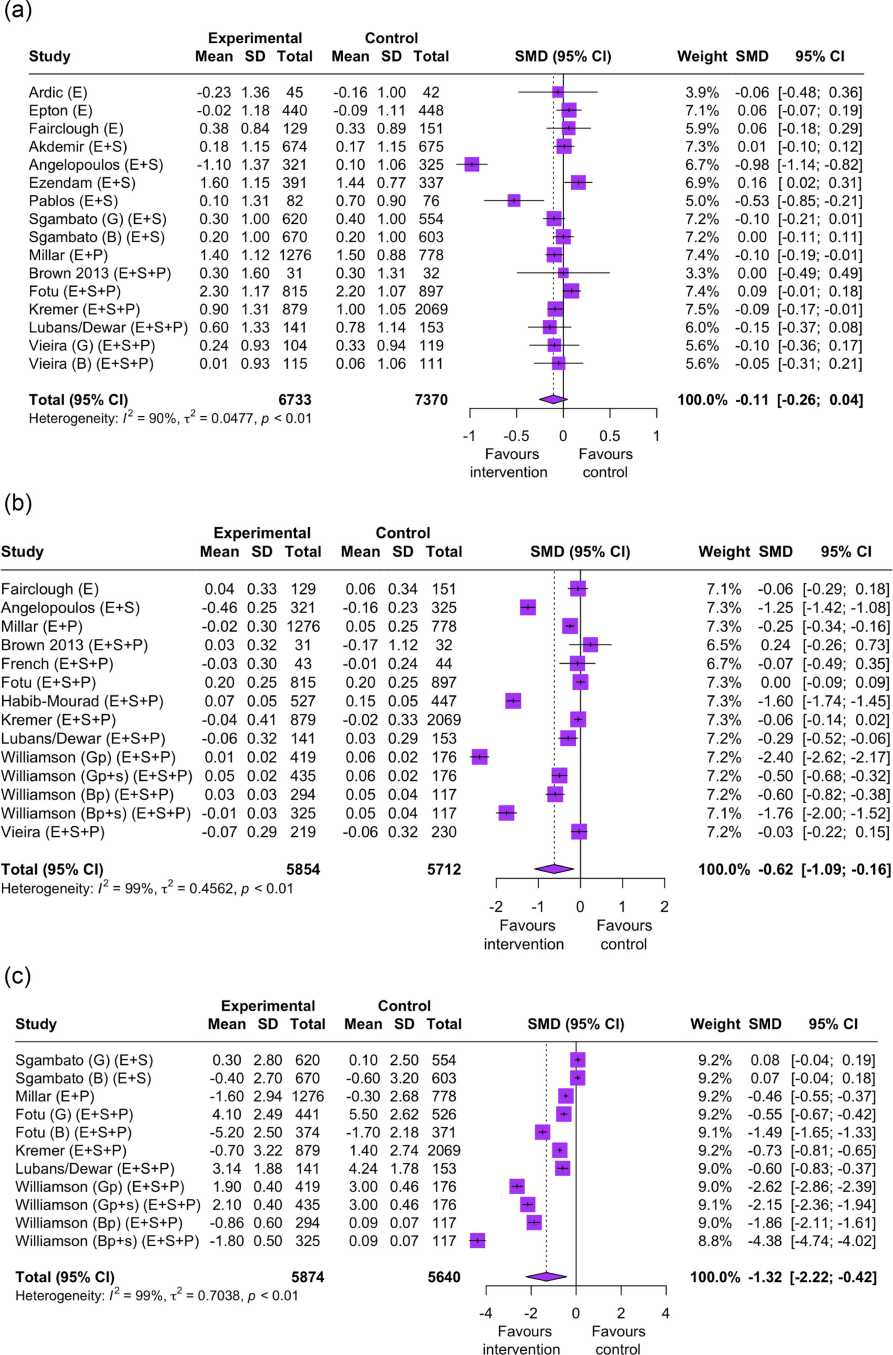
(a) Standardized mean difference (SMD) in body mass index (BMI) for combined diet and physical activity intervention studies (*n* = 14 studies of 16 samples). (b) Standardized mean difference (SMD) in body mass index (BMI) *z* scores for combined diet and physical activity intervention studies (*n* = 11 studies of 14 samples). (c) Standardized mean difference (SMD) in percent body fat for combined diet and physical activity intervention studies (*n* = 6 studies of 11 samples).

Twenty‐eight studies (78%) assessed height and weight and other anthropometric indicators (e.g., waist circumference and body fat percentage) at baseline, with 22 studies (78%) reporting on the effect on changes in at least one outcome at postintervention. Eleven studies (39%) reported a significant change in at least one anthropometric indicator. Fourteen studies (50%), with 16 independent samples, reported data on change in BMI and were included in the meta‐analysis, and eleven studies (39%), with 14 independent samples, reported data on change in BMI *z*‐scores and were included in the meta‐analysis. Studies are reported in the Figures according to the intervention components targeted. There was a nonsignificant reduction in BMI (SMD −0.11 [−0.26, 0.04]; *I* ^*2*^ = 90%; *p* = 0.15) (Figure 2a), but a significant moderate reduction in BMI *z*‐scores (SMD −0.62 [−1.09, −0.16]; *I* ^*2*^ = 99%; *p* = 0.01) (Figure 2b), in favor of the intervention group. Absolute change in BMI and BMI *z*‐score in the intervention groups were 0.45 kg/m^2^ (SD 1.17) and −0.02 (SD 0.21), respectively. This is compared to 0.58 kg/m^2^ (SD 1.02) and 0.02 (SD 0.25) change in BMI and BMI *z*‐score in the comparison groups.

Six studies (21%), with 11 independent samples reported on changes in body fat percentage and were included in the meta‐analysis. Studies are reported in the figures according to the intervention components targeted. There was a significant reduction in body fat percentage in favor of the intervention groups (SMD −1.32 [−2.22, −0.42]; *I* ^*2*^ = 99%; *p* = 0.008). Absolute change in body fat percentage was 0.09% (SD 1.86) for the intervention groups and 1.35% (SD 1.71) for the comparison groups (Figure 2c).

### 3.8 Description of Intervention Features

**FIGURE 3:**
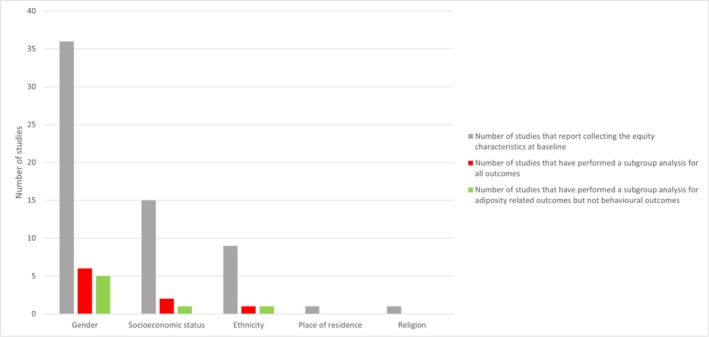
The number of studies that reported equity characteristics at baseline and the number of which reported differential analyses by subgroups.

Figure 3 describes the number of interventions that collected data on equity indicators at baseline and the interventions that conducted analyses according to these indicators. All studies collected data on gender at baseline, with two studies targeting only girls [40, 67]. Six studies (17%) used subgroup analyses to explore the differential effects of gender on all outcomes reported (i.e., behaviors and adiposity if measured) [17, 48, 50, 51, 57, 58], and five (14%) explored the differential effects of gender on adiposity related outcomes only [42, 62, 66, 68, 69]. Differences in the effect of interventions on dietary behaviors, physical activity, and sedentary behaviors according to gender were evident in all studies (Table 3), with little consistency in findings due to the heterogeneity of behaviors targeted and assessed. Similarly, the effect on adiposity‐related outcomes according to gender was mixed with little consistent findings between studies (Table 3 and Figure 3).

Far less studies reported on indicators of socioeconomic position, ethnicity, place of residence and religion, and subgroup analyses were rarely performed (see Figure 4), making it impossible to evaluate the impact of equity characteristics on intervention outcomes.

### 3.9 Intervention Strategies

Supporting File 3 describes details of intervention strategies reported in each of the included studies according to the intervention approach (e.g., educational, education + physical environment), as well as a summary of the effect of each intervention on dietary behaviors, physical activity, sedentary behaviors, and anthropometric indicators. There was little consistency in strategies reported across the different interventions and little consistency in intervention effects by strategies reported and intervention approach.

## 4 Discussion

Adolescence is a time of transition and is often accompanied by radical changes in physical activity and dietary behaviors that can underpin long‐lasting habits and poor health outcomes. The primary aim of this systematic review and meta‐analysis was to synthesize the evidence on the effectiveness of combined dietary and physical activity interventions on changes in dietary behaviors, physical activity, and sedentary behavior in adolescents globally, with a secondary aim of examining the effect of such interventions on adiposity related outcomes where reported. Across 36 studies of adolescents, there was little consistency in interventions in terms of the components, strategies used for behavior change, behaviors targeted and assessed, and the effect of the intervention on behaviors and indicators of adiposity. We found that most interventions were conducted in high‐income countries, included adolescents younger than 15 years of age, and paid little attention to equity issues with very few studies exploring intervention effects by key sociodemographic variables.

To our knowledge, this evidence synthesis provides the most robust evidence to date on the behavioral outcomes of combined dietary and physical activity interventions among adolescents. Dietary behaviors and physical activity have been implicated in the rising prevalence of adolescents living with obesity [70]. However, very few reviews of interventions aiming to prevent obesity among adolescents have provided details on the effect of the interventions on behavioral outcomes ^20.^ This level of information is imperative for policy makers, practitioners, and researchers to better understand how best to change these important behaviors. Five studies were effective at changing both diet and physical activity outcomes targeted; all were school based and targeted fruit and vegetable consumption, dietary fat, diet quality, fried food consumption, total physical activity, and walking. Two were education only, and three were education plus a social environmental component such as homework with parents or parent newsletters. There was little consistency in the remaining studies on changes in diet and physical activity, a moderate effect on reductions in adolescent BMI *z*‐scores and body fat percentage, and no significant reductions in BMI among adolescents. These findings are similar to those shown previously among adolescents ^20.^ Given that obesity is underpinned by multiple health behaviors that exert synergistic effects, it is imperative that we better understand how to effectively target and change multiple health behavior.

In the present review, the large number and the variability in behaviors targeted and the intervention approaches utilized mean that we are limited in our ability to compare interventions and their effects. It might be that changing multiple dietary behaviors, physical activity, and sedentary behaviors at the same time is burdensome for adolescents, and they may lose interest or decide to focus on one behavior. Furthermore, targeting more than one behavior over the course of an intervention period could result in a lack of depth or focus on single behaviors [21, 71]. Indeed, evidence from research comparing single versus multiple health behavior interventions suggest that multiple health behavior interventions are more effective for weight loss, but that single behavior interventions are more effective at changing desired behaviors, albeit with only modest results [72]. In the present review, most studies appeared to have used BCTs for diet and physical activity behaviors that have come from the literature examining correlates and determinants of these individual behaviors. If we are to target multiple health behaviors successfully, research that examines the determinants of clusters or combinations of health behaviors should be drawn upon to identify the most pertinent determinants, which can be mapped onto identifying appropriate BCTs to underpin future interventions.

The present review found that most studies (78%) included in the review had intervention components that were delivered solely in the school setting. While adolescents spend a considerable proportion of their time in the school setting, there has recently been a shift to whole systems approaches when considering changing behaviors such as diet [73] and/or physical activity [11]. The mixed effectiveness and the inconsistencies in the findings across the categories of intervention approaches in the present review could be that, regardless of intervention approach (i.e., educational and/or social environmental strategies), these, often school based, interventions have targeted individual (personal) behavior change. Adolescents nowadays operate in systems that are highly digital and driven by proximal (e.g., parents, peers, and wider community) and distal (e.g., cultural norms, customs, and policies) influences [74], and thus, for interventions to have the greatest effect on changing physical activity, diet, and adiposity indicators, there needs to be a shift in focus to intervening in parts or the whole of adolescents' system where the greatest impact can be achieved. The context and lived experiences of adolescents are key drivers of behavior, and thus, involving adolescents as active partners in the focus, design, and implementation of interventions to change behavior should be a priority to ensure that the strategies and components are current and user focused.

All interventions in this review included educational strategies either as the sole component or as part of multicomponent intervention approaches. Educational strategies such as efforts to increase knowledge or to teach young people about health risks of behaviors, for example, come from decision making theories proposing that increased knowledge will lead to positive behavior change. However, educational approaches for behavior change with adolescents have not been established to be as effective as educational approaches targeting younger children [75]. It has been argued that interventions focusing on providing knowledge or self‐regulation skills are ignoring or fighting against the drivers for engaging in these “problem” behaviors in the first place [75]. Educating adolescents on the importance of physical activity and choosing healthy foods is unlikely to be effective without considering the wider systems in which adolescents operate, which may or may not be supportive of positive health behaviors. While most interventions included in this review utilized social and or physical environmental strategies (i.e., multicomponent) in addition to the educational strategies, there appeared to be no clear intervention effects when stratified by broad intervention components (i.e., educational versus educational + social environmental), which could be partly explained by the diverse BCTs used within the different components, or differences in the characteristics of the intervention (e.g., implementation modality, dose, duration, and fidelity). Such heterogeneity, alongside the lack of detail and reporting of intervention approaches, makes it challenging to identify the specific components within the combined diet and physical activity interventions that contribute to the lack of consistency and limited effectiveness of interventions at changing both dietary and physical activity behaviors. This poses both challenges and opportunities for further research. There is a need to understand what works and why, and much of this could be uncovered from more robust reporting of implementation and process evaluations, as well as standardization of measurement and reporting. Furthermore, to truly change complex behaviors that have the capacity to influence health, there is a need for a whole systems approach, targeting the multiple settings that children and young people operate in, that begins in early childhood to establish foundational healthy habits [38, 74]. Approaches that include multiple stakeholders from a range of sectors across communities are needed to create lasting environmental and societal changes that support healthier behaviors across the lifespan.

The behaviors that were targeted were not consistent across interventions but included both the reduction in the consumption of unfavorable foods/drinks (e.g., skipping breakfast, sugar sweetened beverages), the increase in favorable foods/drinks (e.g., fruit and vegetable consumption), and increase in domains of physical activity (e.g., active travel). The strategies used in the interventions ranged considerably, with common BCTs [44] included goal setting, feedback, monitoring, knowledge, and modeling. In some papers, unclear descriptions precluded specific identification of the BCTs utilized. Little attention was given, within the studies included in this review, to implementation processes. Given that each intervention included in this review targeted multiple dietary and physical activity behaviors, utilized several behavior change strategies and many were targeting more than one component (i.e., complex interventions), it is hard to say whether the setting, components, and strategies are not effective at changing behaviors, or whether there is particularly poor fidelity across these interventions. There was no mention of implementation strategies across the included studies. Understanding the implementation of complex interventions is critical for many reasons including maximizing effectiveness, identifying key barriers and facilitators to successful implementation, and improving the adaptability of interventions shown to be effective in certain settings. A better understanding of how complex interventions targeting multiple health behaviors are implemented will have significant implications for optimizing health behaviors and health outcomes by improving the efficiency of interventions, making interventions more adaptable, sustainable, and scalable, and can inform future research and policy decisions and strategies to enable the creation of supportive systems.

A clear gap in the evidence from this review is the information coming from low‐ and MICs. For instance, only one study has generated evidence from the African region, and there is no evidence identified for low‐income countries. This review has shown a heterogeneity of findings across and within the components targeted and strategies used in interventions, and a variety of findings in relation to sociodemographic inequities, highlighting the potential importance of future work taking a realist perspective [47] in understanding the effectiveness of these types of interventions. Evidence on what works for whom and in what context could potentially be important in helping to interpret the heterogeneity in results seen across these types of study. The context of low‐ and MICs is different in many ways to high income countries (e.g., differences in climatic conditions, active transport, food security, poverty, and cultural differences), and evidence is needed for what works in these different types of physical and social environments in relation to improving physical activity, sedentary behavior, and dietary outcomes to understand what has the potential to work to change behaviors and reduce obesity in the context of low‐middle income countries.

### 4.1 Strengths and Limitations

There are limitations to the present review, some of which are due to gaps in the literature itself. The review has revealed a bias in the geographical regions represented, with no evidence from low‐income countries and limited evidence from MICs. Also, the majority of the studies from the high‐income countries came from Europe (44%) and the United States (36%), with many countries/regions not represented in the literature. Studies were heterogenous in character (e.g., components, strategies used, and behaviors targeted and assessed), making it challenging to assess the overall consistency of effectiveness. Few interventions targeted and assessed the same combination of dietary and physical activity behaviors, thus limiting the possibility of drawing strong conclusions on the effectiveness of interventions on specific behavioral outcomes. Furthermore, due to the lack of clarity in the papers, it was not possible to map the specific BCTs used in each intervention. RoB in studies was high and most relied on reported behaviors. Self‐report tools should ideally be replaced or augmented with objective measures, such as accelerometers, to minimize errors caused by recall bias and social desirability, which often result in inaccurate data [49]. Furthermore, and importantly, when more objective tools are used, it is important that rigor and a level of standardization are applied in the methods of deployment and data reduction [53].

Key strengths of this review include the robust search and systematic approach to synthesizing 36 published studies, the inclusion of controlled interventions which can provide greater certainty of evidence, the focus on adolescents and the combined dietary and physical activity interventions, the clear definitions of the effect of the interventions on all dietary and physical activity behaviors reported in studies, and the extraction of all utilized intervention strategies and equity effects providing a comprehensive overview of the published literature and highlighting gaps to be addressed in future research. Furthermore, no restrictions were placed on the searches for the review in terms of language, countries, or publication date.

## 5 Conclusion

The evidence for interventions targeting both dietary and physical activity behaviors and their effect on change in behavior and adiposity in adolescents is largely inconsistent. The positive findings of some studies suggests that there is potential to improve these lifestyle behaviors and associated adiposity outcomes in adolescents in some contexts. However, the current evidence is focussed on high income countries with little consideration given to potential inequities in the effects of interventions even within those countries. This results in a lack of understanding in the evidence of what works for whom across a range of contexts. Further work is needed to understand the implementation process of what are often complex interventions, and how these can be optimized in contexts that are diverse and multifaceted.

## Author Contributions

N.P. and L.B.S. conceived the study, N.P. carried out the design, and N.P. and R.P. developed the search strategy. B.B., A.P.S., N.P., and R.P. conducted the searches, screening, data extraction and RoB. N.P., A.K., and R.P. carried out the synthesis, and N.P. interpreted the results and drafted the manuscript. P.G. and L.B.S. provided methodological input and advised on the interpretation of the results. All authors assisted in the editing of the manuscript and associated tables and figures. All authors read and approved the final manuscript.

## Ethics Statement

The authors have nothing to report.

## Consent

The authors have nothing to report.

## Conflicts of Interest

The authors declare no conflicts of interest.

## Supporting information




**Data S1.** Search strategy for PubMED.

**Data S2.** Final to populate.

**Data S3.** Detail of components and strategies utilised in intervention studies, and the effect of intervention on dietary behaviours, physical activity, sedentary behaviour and indicators of adiposity.

**Data S4.** Overall risk of bias from randomised controlled trials.

## Data Availability

All data generated or analyzed during this study are included in this published article and its supporting information files.
