# Supplementary material for: The Effectiveness of Combined Dietary and Physical Activity Interventions for Improving Dietary Behaviors, Physical Activity, and Adiposity Outcomes in Adolescents Globally: A Systematic Review and Meta‐Analysis
Source: Obes Rev. 2025 May 20;26(9):e13940. doi: 10.1111/obr.13940 (PMC12318910; doi:10.1111/obr.13940)
Supplement: Supplementary file 1 — Data S1. Search strategy for PubMED. [file OBR-26-e13940-s002.pdf]

Additional File 1. Search strategy for PubMED

Search number	Query
8	(#2 AND #3 AND #4 AND #5) not (#6 or #7)
7	"pre-school"[Title/Abstract] OR "pre-schooler*"[Title/Abstract] OR "preschool"[Title/Abstract]
6	"editorial"[Publication Type] OR "comment"[Publication Type] OR "news"[Publication Type] OR "letter"[Publication Type] OR "review"[Publication Type] OR "systematic review"[Publication Type] OR "systematic review"[Title/Abstract] OR "meta-analysis"[Publication Type] OR "meta-analysis"[Title/Abstract] OR "meta-analyses"[Title/Abstract] OR "retracted publication"[Publication Type] OR "retraction of publication"[Publication Type] OR "retraction of publication"[Title/Abstract] OR "retraction notice"[Title/Abstract] OR "qualitative"[Title/Abstract] OR "cross-sectional"[Title/Abstract]
5	"diet*"[Title/Abstract] OR "nutrition*"[Title/Abstract] OR "eating"[Title/Abstract] OR "dietary behaviour*"[Title/Abstract] OR "dietary behavior"[Title/Abstract] OR "eating behaviour*"[Title/Abstract] OR "eating behavior*"[Title/Abstract] OR "fruit"[Title/Abstract] OR "vegetables"[Title/Abstract] OR "energy-dense"[Title/Abstract]
4	"sports"[MeSH Terms] OR "exercise"[MeSH Terms] OR "physical activities"[Title/Abstract] OR "energy expenditure"[Title/Abstract] OR "sports"[Title/Abstract] OR "active travel"[Title/Abstract] OR "walking"[Title/Abstract] OR "cycling"[Title/Abstract] OR "dancing"[Title/Abstract]
3	"intervention"[Title/Abstract] OR "trial"[Title/Abstract] OR "randomised"[Title/Abstract] OR "randomized"[Title/Abstract] OR "controlled"[Title/Abstract] OR "comparison"[Title/Abstract] OR "experiment"[Title/Abstract] OR "quasi-experimental"[Title/Abstract] OR "cluster"[Title/Abstract] OR "programme"[Title/Abstract] OR "program"[Title/Abstract] OR "scheme"[Title/Abstract]
2	"child*"[MeSH Terms] OR "adolescent"[MeSH Terms] OR "adolescence"[Title/Abstract] OR "teen*"[Title/Abstract] OR "young people"[Title/Abstract] OR "youth*"[Title/Abstract] OR "boy"[Title/Abstract] OR "girl"[Title/Abstract] OR "school aged"[Title/Abstract]
1	"child*"[MeSH Terms] OR "adolescent"[MeSH Terms] OR "adolescence"[Title/Abstract] OR "teen*"[Title/Abstract] OR "young people"[Title/Abstract] OR "youth*"[Title/Abstract] OR "boy"[Title/Abstract] OR "girl"[Title/Abstract] OR "school aged"[Title/Abstract]
