# Supplementary material for: The Effectiveness of Combined Dietary and Physical Activity Interventions for Improving Dietary Behaviors, Physical Activity, and Adiposity Outcomes in Adolescents Globally: A Systematic Review and Meta‐Analysis
Source: Obes Rev. 2025 May 20;26(9):e13940. doi: 10.1111/obr.13940 (PMC12318910; doi:10.1111/obr.13940)
Supplement: Supplementary file 3 — Data S3. Detail of components and strategies utilised in intervention studies, and the effect of intervention on dietary behaviours, physical activity, sedentary behaviour and indicators of adiposity. [file OBR-26-e13940-s004.pdf]

Supplementary File 3: Detail of components and strategies utilised in intervention studies, and the effect of intervention on dietary behaviours, physical activity, sedentary behaviour and indicators of adiposity.

Study	Strategies	Effect on dietary behaviours		Effect on physical activity	Effect on sedentary behaviours	Effect on indicators of adiposity
		UF	F			
**Educational components only**						
Ardic et al. 2017	Self-esteem, positive thinking, self-talk<br>Goal setting<br>Problem solving<br>Emotional and behavioural regulation<br>Barriers to goal progression and overcoming barriers<br>Stress and coping<br>Effective communication: personality and communication styles	NM	↑*	↑*	NA	mixed
Contento et al. 2010	Goal setting<br>Knowledge<br>Outcome expectations<br>Goal intentions<br>Role play<br>Science activities<br>Monitoring<br>Competence and autonomy support	↓*	0	↑*	↓*	NM
Epton et al. 2014	Self-affirmation<br>Theory-based messages<br>Implementation intentions	NM	0	0	0	0
Fairclough et al. 2013	Knowledge<br>Mapping opportunities<br>Monitoring<br>Goal setting	NM	0	0	0	mixed

Francis et al. 2010 & Nichols et al. 2014	Cognitive / knowledge<br>Affective<br>Psychomotor<br>PA demonstrations	↓*	0	0	0	NR
Frenn et al. 2005	Consciousness raising<br>Self-evaluation<br>Feedback<br>Decisional balance	↓*	NM	↑*	NM	NM
Rutsztein et al.2023	Active participation of adolescents promoted through activities, videos and games with attractive and entertaining content.<br>Critical analysis of media.<br>Homework.	↓*	NM	0	NM	NR
Weigensberg et al. 2021	Didactic and experiential education relating to healthy eating and physical activity	0	0	0	0	NM
**Educational and social environmental components**						
Akdemir et al. 2017	Educational activities to adolescents on healthy nutrition and active lifestyle as well as the causes of and preventive strategies for obesity.<br>Education to families and a healthy-nutrition brochure for families.	↓*	↑*	↑*	↓*	mixed
Angelopoulos et al. 2009	Knowledge – active learning<br>Modelling<br>Enactment<br>Guided practice<br>Self-esteem – positive thinking, self-talk<br>Goal setting<br>Problem solving<br>Self-monitoring<br>Mobilizing social support<br>Arguments<br>Direct experience	↓*	mixed	↑*	NM	mixed

	Self-re-evaluation<br>Environment re-evaluation					
Champion et al. 2023	Health4Life, an eHealth MHBC programme that provides students with simultaneous education about the Big 6, the relationships between them, and their relationship with mental health.<br>Online cartoon modules that use co-designed storylines about a group of teenagers and principles of social influence to impart evidence-based information about the Big 6.<br>Web-based, targeted feedback about adherence to national health guidelines and optional online and teacher-delivered activities.<br>Cognitive-behavioural and motivation-enhancement techniques to help develop students' coping strategies and skills to facilitate healthy habits	0	NM	0	0	NM
Ezendam et al. 2012	Feedback / normative feedback<br>Intention formation<br>Decisional balance information<br>Barrier identification<br>Instruction<br>Organise social support	↓*	mixed	mixed	0	0
Jemmott III et al. 2019	Games<br>Brainstorming<br>Role-play<br>Group discussion<br>Comic books/stories<br>Knowledge/information<br>Addressed barriers to adherence<br>Homework to enlist parents to support changes	↓*	↓*	mixed	NM	NM
Pablos et al. 2018	Education/information<br>Exercise sessions<br>Themed games	NM	0	0	0	mixed

	Parent information<br>Homework activities					
Patrick et al. 2006	Feedback<br>Progress plans<br>Parent information<br>Counselling<br>Role play<br>Identify benefits of change<br>Goal setting<br>Identify social support<br>Reduce barriers<br>Problem solving<br>Relapse prevention<br>Skills	0	mixed	mixed	↓*	0
Prieto Zambrano et al. 2021	Knowledge / information for children and parents<br>Play activities	0	0	0	NM	NR
Sevil et al. 2019	Need-supportive teacher-training programme, (i.e., autonomy, competence, and relatedness support)<br>Support<br>Role modelling<br>increasing knowledge and awareness of health-related recommendations<br>educating in optimal time distribution, allowing reallocations between PA, ST, and SD<br>Empowering adolescents to manage health decisions.<br>Parents were provided with healthy lifestyle and autonomy-supportive strategies (e.g., adolescents were provided with choices and opportunities to participate in PA)<br>Sports activities/events	↓*	↑*	↑*	mixed	NR
Sgambato et al. 2019	Educational games, group debates and culinary classes through schools	NM	mixed	↑*	NM	Mixed

	Teacher training.<br>Family activities.					
Spiegel et al. 2006	Directed-reflective journaling.<br>Class discussion<br>Considered beliefs of themselves and others<br>Attitudes<br>Behavioural skills<br>Self-analysis of behavioural intent<br>Subjective norms<br>Skills<br>Role play<br>Journalling<br>Goal setting<br>Parent component	NM	↑*	↑*	NM	↓*
Thi Nguyen et al.	Peer-education,<br>Peer support,<br>Diet monitoring	mixed	0	0	0	NR
**Educational and physical environmental components**						
Barbosa-Filho et al. 2019	Opportunities<br>Equipment<br>Education<br>Environmental changes	0	0	↑*	↑*	NM
Chawla et al. 2017	Environmental changes<br>Life skills: education on obesity consequences, healthy eating guidance, nutrition labelling, physical activity, healthy choices and growing vegetables.	0	0	0	0	0
Millar et al. 2011	Education/information<br>Capacity building – training school staff and students<br>Awareness – social marketing<br>Evaluating<br>Increased access / opportunities for PA<br>Promoting water and reducing access to soft drinks	0	0	↑*	mixed	mixed

	Increased healthfulness of school foods – traffic light system					
Tarro et al. 2019	Training adolescent creative changers<br>Activities and games around knowledge<br>Opportunities to try raw fruits/veg<br>Opportunities to try new PA through games	mixed	0	0	mixed	NM
**Educational, social and physical environmental components**						
Aceves-Martins et al. 2022	Training of adolescent challenge creators (ACCs)<br>Social marketing strategies: customer or participant orientation, behaviour, theory, insight, exchange, competition, segmentation.<br>Designing classroom and playground activities to increase physical activity and healthy eating.<br>Campaign exhibitions<br>Photographs	NM	0	mixed	0	0
Baltaci et al. 2022	Skill-building activities together and separately in parent only, youth only, or parent/youth joint activities.<br>food preparation, eating a meal together, parenting skills education, nutrition/physical activity education (together and separately), and physical activity (together).<br>Education for parents focused on parenting skills related to parent child interactions and food- and activity-related parenting practices.<br>Education for youth focused on EBRBs and building strong family communication and connections.<br>Parent and youth joint activities involved explanations of basic nutrition and physical activity concepts and hands-on practice/discussion based on their experiences.	0	0	0	0	0

	Highlighted healthy eating and physical activity and their associations with overall health instead of weight loss.<br>Discussion guide handouts and take-home activity sheets.					
Brown et al. 2013	Cultural components, addressing youth's knowledge of and access to healthy food, including hands-on interactive learning activities and using a group format to deliver the intervention.<br>Emphasis on traditional activities (such as berry picking, horseback riding, dancing, hunting, hiking, and camping), use of storytelling and native language to convey information, and participation of elders.<br>Hands-on interactive activities included preparing and tasting healthy snacks: learning about and participating in native games: keeping and discussing weekly activity and nutrition diaries: and group games for finding hidden calories in fast-food menu items and convenience store food items.	0	NM	0	0	0
Brown et al. 2014	Information/knowledge sharing<br>Consciousness-raising<br>Self-monitoring<br>Enlisting social support<br>Outcome expectations<br>Barrier identification<br>Action planning<br>Coping planning<br>Goal setting<br>Social integration and support<br>Time management<br>Novel experiences<br>Planning<br>Rewards	NM	0	↑*	NM	NM

	Environmental changes					
Fotu et al. 2011	Social marketing approaches<br>Community capacity building and grass roots activities to promote healthy behaviours, including eating breakfast, increasing water, fresh fruit and vegetable consumption, participation in organized sports and physical activity during and after school, and reducing sweet drink consumption and sedentary activities	mixed	mixed	↓*	0	mixed
French et al. 2011	Goal setting/tracking<br>Self-monitoring<br>Positive reinforcement<br>Home activities: newsletters, reinforcing messages, homework, incentives.<br>Digital scales for weight monitoring.<br>TV limiting device.<br>Support phone calls and emails<br>Problem solving	0	↑*	0	0	0
Habib-Mourad et al. 2020	Information/knowledge<br>Parent component – healthy home environment<br>Environmental change<br>PA sessions	↓*	↑*	0	NM	0
Haerens et al. 2006	Information<br>Increased opportunities for PA<br>Increased access to FV<br>Physical environmental changes – water fountains<br>Equipment<br>Fitness tests<br>Schools encouraged active transportation<br>Tailored feedback on intentions, attitudes, self-efficacy, social support, knowledge, benefits and barriers related to physical activity<br>Tailored advice	mixed	0	mixed	0	NR

	Parental involvement: meetings and newsletters, information folder					
Kremer et al. 2011	Information/educational<br>Environmental changes – increase access.<br>Environmental audit<br>Posters<br>Training of educators<br>Awareness programme for parents and community<br>Skill development	0	mixed	0	mixed	mixed
Lubans et al. 2012 & Dewar et al. 2013	Enhanced school sport sessions<br>Lunchtime physical activity sessions<br>Nutrition workshops<br>Interactive educational seminars<br>Pedometers for self-monitoring<br>Student handbooks<br>Parent newsletters, and text messages to reinforce and encourage targeted health behaviours.	0	NM	0	↓*	0
Vieira et al. 2021	Encouraging parents to increase F&V availability at home and support children's behaviour changes.<br>Increased access and availability of FV at school<br>The core constructs of TTM (processes of change, decision balance, self-efficacy) were also applied for supporting their progress across stages and to engage them to participate actively in order to increase knowledge and nutritional literacy, to develop competencies for decision-making on healthy choices.<br>Cognitive, attitudinal, and behavioural strategies based on the TTM (not told specific strategies)	mixed	0	↑*	0	mixed
Williamson et al. 2012	Modified environmental cues related to healthy eating and physical activity.<br>Modified the cafeteria food service program.<br>modified the physical education programs.<br>Modified content of vending machines.	0	NM	0	0	0

	Classroom instruction.<br>Internet counselling and asynchronous (email) communications for children and their parents					

UF: unfavourable dietary behaviours: F: favourable dietary behaviours: NM: not measured: NR: not reported: mixed: some positive, some negative and some null findings
