# Supplementary material for: The Effectiveness of Combined Dietary and Physical Activity Interventions for Improving Dietary Behaviors, Physical Activity, and Adiposity Outcomes in Adolescents Globally: A Systematic Review and Meta‐Analysis
Source: Obes Rev. 2025 May 20;26(9):e13940. doi: 10.1111/obr.13940 (PMC12318910; doi:10.1111/obr.13940)
Supplement: Supplementary file 4 — Data S4. Overall risk of bias from randomised controlled trials. [file OBR-26-e13940-s003.pdf]

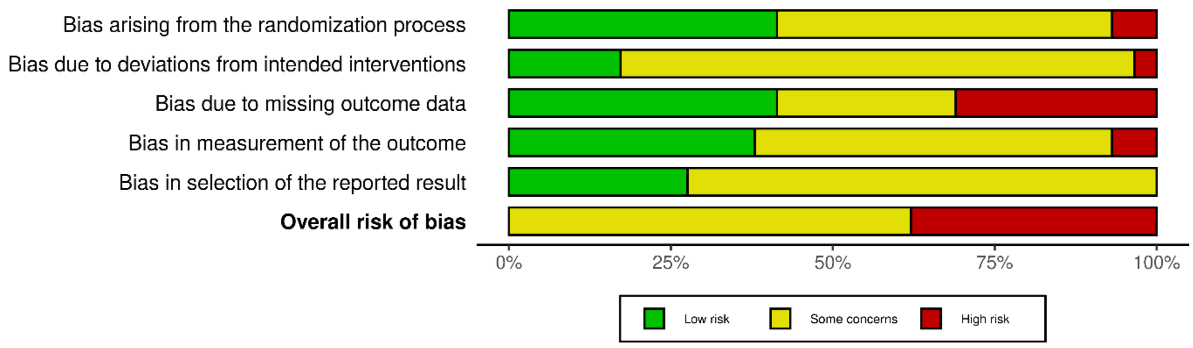

Additional Figure 2a. Overall risk of bias from randomised controlled trials

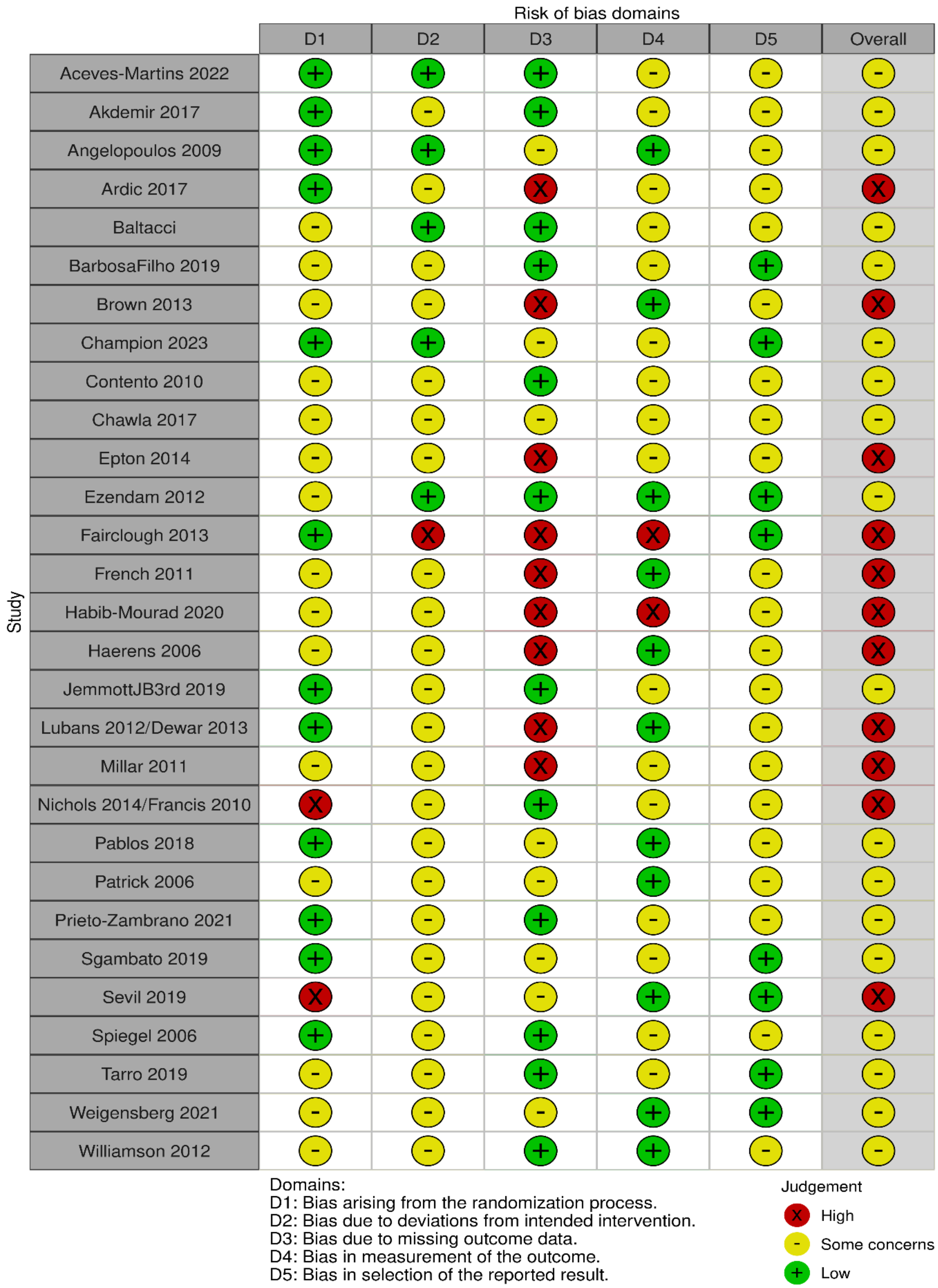

Additional Figure 2b. Risk of bias domains for included randomised controlled trials

Figure 3

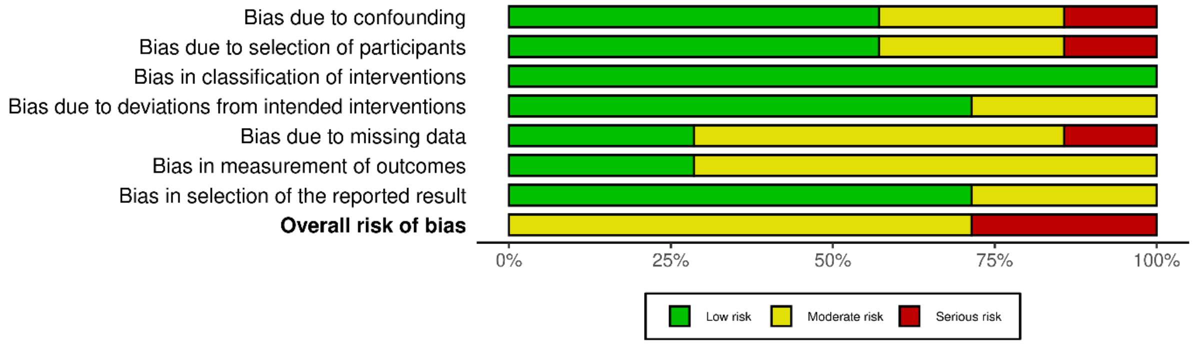

Additional Figure 2c. Risk of bias for included non-randomised controlled trials

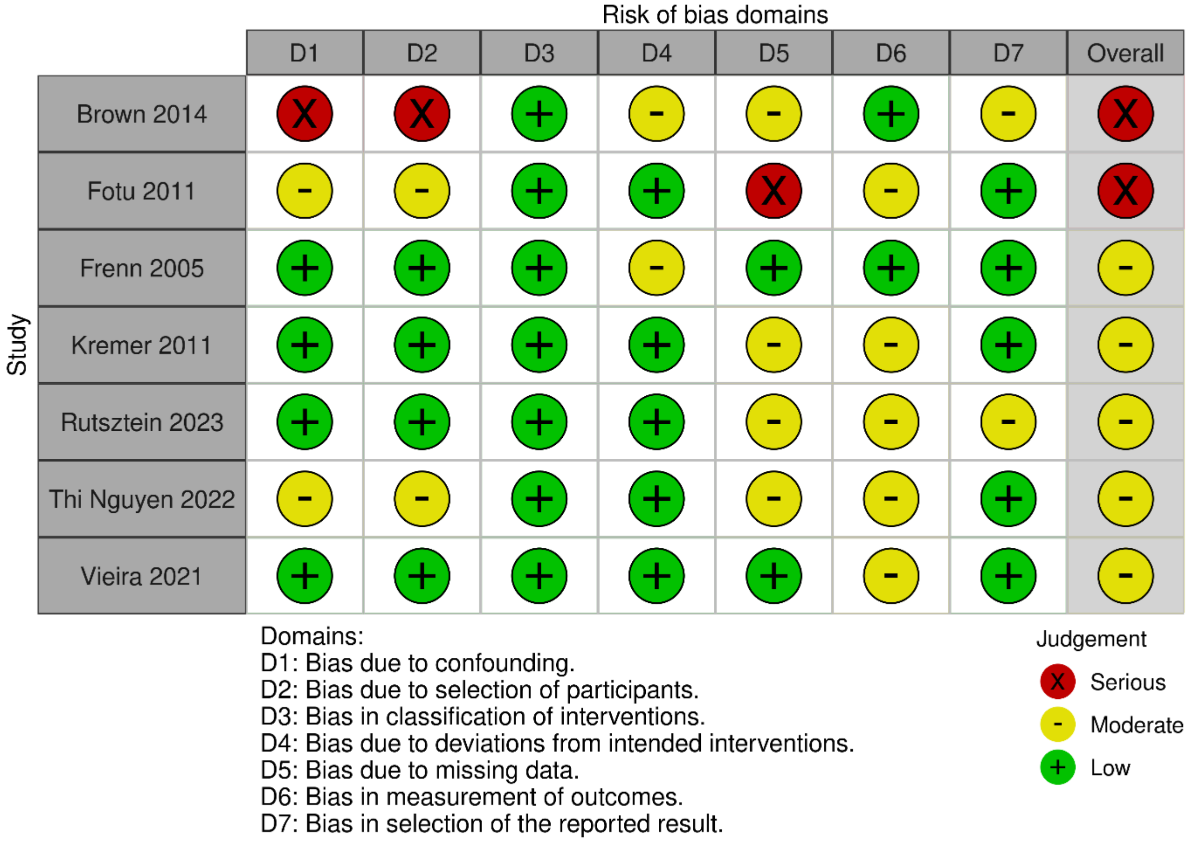

Additional Figure 2d. Risk of bias domains for non-randomised controlled trials
